# Characterization of Conformational Ensembles of Protonated N-glycans in the Gas-Phase

**DOI:** 10.1038/s41598-018-20012-0

**Published:** 2018-01-26

**Authors:** Suyong Re, Shigehisa Watabe, Wataru Nishima, Eiro Muneyuki, Yoshiki Yamaguchi, Alexander D. MacKerell, Yuji Sugita

**Affiliations:** 1 RIKEN Theoretical Molecular Science Laboratory, 2-1 Hirosawa, Wako, Saitama, 351-0198 Japan; 2 RIKEN iTHES, 2-1 Hirosawa, Wako Saitama, 351-0198 Japan; 3 Graduate School of Science and Engineering, Chuo University, 1-13-27, Kasuga, Bunkyo-ku, Tokyo, 112-8551 Japan; 4 Structural Glycobiology Team, Systems Glycobiology Research Group, RIKEN Global Research Cluster, 2-1 Hirosawa, Wako Saitama, 351-0198 Japan; 5 Department of Pharmaceutical Sciences, School of Pharmacy, University of Maryland, Baltimore, Maryland, 21201 USA

## Abstract

Ion mobility mass spectrometry (IM-MS) is a technique capable of investigating structural changes of biomolecules based on their collision cross section (CCS). Recent advances in IM-MS allow us to separate carbohydrate isomers with subtle conformational differences, but the relationship between CCS and atomic structure remains elusive. Here, we characterize conformational ensembles of gas-phase N-glycans under the electrospray ionization condition using molecular dynamics simulations with enhanced sampling. We show that the separation of CCSs between isomers reflects folding features of N-glycans, which are determined both by chemical compositions and protonation states. Providing a physicochemical basis of CCS for N-glycans helps not only to interpret IM-MS measurements but also to estimate CCSs of complex glycans.

## Introduction

Carbohydrates are ubiquitous components of living organisms. As over 50% of eukaryotic proteins and most of membrane proteins are glycosylated^1^, their roles in biological processes, ranging from protein folding to biomolecular recognition events, have received considerable attention^2–4^. The biological activity of a glycoconjugate is often affected by a subtle change in a carbohydrate structure^5^. Carbohydrates have multiple linkage points and take branched structures. In addition, many rotamers arise along flexible linkages. The hydroxyl groups act as both donor and acceptor for hydrogen (H-) bonds, potentially forming a complex network of interactions to stabilize various conformations. Carbohydrates are commonly a complex mixture of various isomers, each having multiple conformations, and analysis of their structures in solution is extremely difficult^6^.

Ion mobility mass spectrometry (IM-MS) is an analytical method increasingly used for obtaining information of conformational changes in polyatomic ions, such as unfolding or aggregation^7–9^. This method separates gas-phase ions based on the collision cross section (CCS) that reflects their sizes and shapes. IM-MS is capable of separating stereoisomers with identical mass but different conformations^10^. The method has been increasingly applied for structure determination of carbohydrates^11–14^. Recently, highly sensitive techniques have been reported for separating diastereomers of synthetic carbohydrates as well as those of glycoconjugates^15–21^. For instance, epimeric monosaccharides, which differ in chirality of only one hydroxyl group, can be differentiated^17^. However, conformational diversity as well as chemical heterogeneity of carbohydrates raises questions about the interpretation of IM-MS data. First, the experimentally derived CCS represents either a single species or multiple isomers and/or conformers. Second, the ionization of samples (e.g. protonation or sodiation) modifies their conformational ensemble. To date, limited investigations have been undertaken on gas-phase conformations at a finite temperature, while many studies have focused on solution conformations^22^. Thus, the determinants of CCSs of carbohydrates still remain largely unknown.

We previously reported a combined IM-MS and molecular dynamics (MD) simulation study on the identification of isomeric N-glycan structures^23^. Such a combined approach enabled us to link the measured CCS with structures at atomic resolution^7,24,25^. We measured the drift times of ten pyridylamino (PA)-glycans, including isomeric forms, in the presence of N_2_ gas by using traveling wave ion mobility mass spectrometry. The PA group was introduced to enhance detection sensitivity. Doubly protonated PA-glycans ([M + 2H]^2+^) predominated. Then, CCS values were derived by calibration with polyalanine, and different values were obtained for different isomers. Finally, we performed replica-exchange molecular dynamics (REMD) simulations^26^ of isomeric N-glycans. The REMD simulation facilitated transitions between rotameric isomers that rarely occur in conventional MD simulations, allowing us to obtain conformational ensembles with high statistical accuracy. CCS values were calculated using the MOBCAL program^27,28^. The CCS is commonly calculated for a few selected low-energy conformers and compared with experimentally obtained values^29,30^. Our approach was different in that we obtained a population-weighted CCS based on the ensemble of conformations. We showed that the isomeric N-glycans fold to unique shapes, giving distinct CCS values.

**Figure 1:**
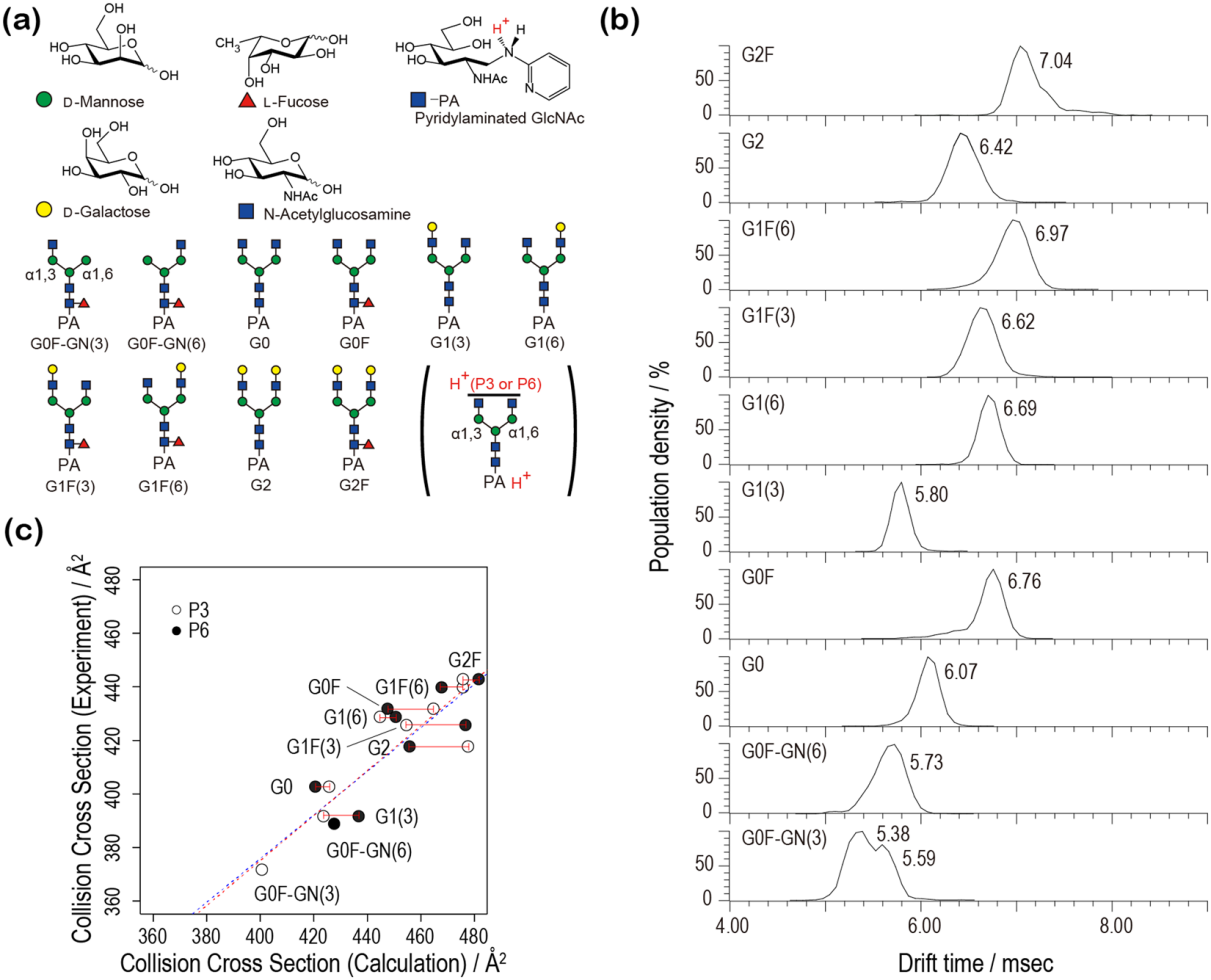
(**a**) Symbolic representation of the ten PA-glycans studied. (**b**) Experimentally observed arrival time distributions of the ten PA-glycans^23^. (**c**) Plot of calculated Collision Cross Sections (CCSs) in N_2_ gas against experimental values obtained in N_2_ gas. P3 and P6 indicate the different protonation states (P3: H^+^ at α1-3 (white) and P6: H^+^ at α1-6 branch (black)) (Dotted lines: regression lines with the correlation coefficient of 0.90 (P3, blue) and 0.89 (P6, red), respectively).

In the present study, we computationally investigated CCS determinants of N-glycans to predict CCSs from the structural information. To this end, we extended the previous simulation study in several ways. First, the target system is expanded to ten pyridylamino (PA)-glycans (Fig. 1a). Second, we explicitly model doubly protonated PA-glycans ([M + 2H]^2+^) to directly compare with experiment under the electrospray ionization condition^23^. The protonation sites are not known experimentally. Considering that the amide group is more readily protonated than the hydroxyl group^31^ and that protons electrostatically repel each other, we built two models with H^+^ on PA and on the distal N-acetylglucosamine (GlcNAc) of either the α1-3 (P3) or α1-6 branch (P6). The consideration of protonation states adds further complexity to the calculations. As force field parameters of protonated species are not available in CHARMM36^32^, which is used to perform MD simulations, the missing parameters for protonated PA and GlcNAc were specially developed for the present study. Third, we calculate CCS values in N_2_ drift gas using the trajectory method^27,33^, while a simplest projection approximation assuming He drift gas condition was used in our previous study^23^. We use the modified version of the MOBCAL program and newly parametrized N_2_-based trajectory method algorithm^34^. The Replica-Exchange INter-face (REIN) is used for the REMD simulations^35^. All analyses were done using the trajectories at 300 K. Details of modeling are given in Supplementary information.

## Results and Discussion

### Experimental collision cross section (CCS) for 10 PA-glycans

The studied system (Fig. 1a) covers representative N-glycan modifications, e.g. core-fucosylation and/or galactosylation, which are known to modulate protein functions. We previously reported the drift time of doubly protonated PA-glycans in N_2_ gas by using traveling wave ion mobility mass spectrometry (Fig. 1b)^23^. In the present work, we recalibrated CCS values of doubly protonated PA-glycans using the absolute CCS values of doubly protonated polyalanine^36^ in N_2_ gas, rather than the singly protonated species used before^23^. They are listed in Table 1. Five points (*n* = 11, 12, 13, 14, and 15) were used for the calibration since the polyalanine with *n* > 15 becomes triply charged. A good linearity was found for the drift time and CCS of polyalanine as shown in Table 1. We thus consider that the standard curve can be used for the calculation of glycan CCS.

**Table 1:** Experimentally measured drift times and estimated collision cross sections (CCSs) in N_2_ gas for ten doubly protonated PA-glycans. CCS values of doubly protonated polyalanines used for the calibration are also shown for comparison.

***Glycan***	ion species	m/z	drift time (msec.)^a^	CCS in N_2_ (Å^2^)
G0F-GN(3)	[M + 2H]^2+^	669.8	5.31	371
G0F-GN(6)	[M + 2H]^2+^	669.8	5.73	388
G0	[M + 2H]^2+^	698.3	6.07	402
G0F	[M + 2H]^2+^	771.3	6.76	431
G1(3)	[M + 2H]^2+^	779.3	5.80	391
G1(6)	[M + 2H]^2+^	779.3	6.69	428
G1F(3)	[M + 2H]^2+^	852.3	6.62	425
G1F(6)	[M + 2H]^2+^	852.3	6.97	440
G2	[M + 2H]^2+^	860.3	6.42	417
G2F	[M + 2H]^2+^	933.4	7.04	442
***Polyalanine (n)*** ^**b)**^				
10	[M + 2H]^2+^	365.0	3.24	not available
11	[M + 2H]^2+^	400.5	3.52	296
12	[M + 2H]^2+^	436.0	3.79	309
13	[M + 2H]^2+^	471.5	4.07	320
14	[M + 2H]^2+^	507.0	4.35	333
15	[M + 2H]^2+^	542.5	4.69	344

^a^Ref.^23^.

^b^Ref.^36^. A good linearity was found for the drift time (*t*) and CCS of polyalanine (CCS = 41.3  × *t* + 152 with R^2^ = 0.996).

#### Calculated CCS

**Figure 2:**
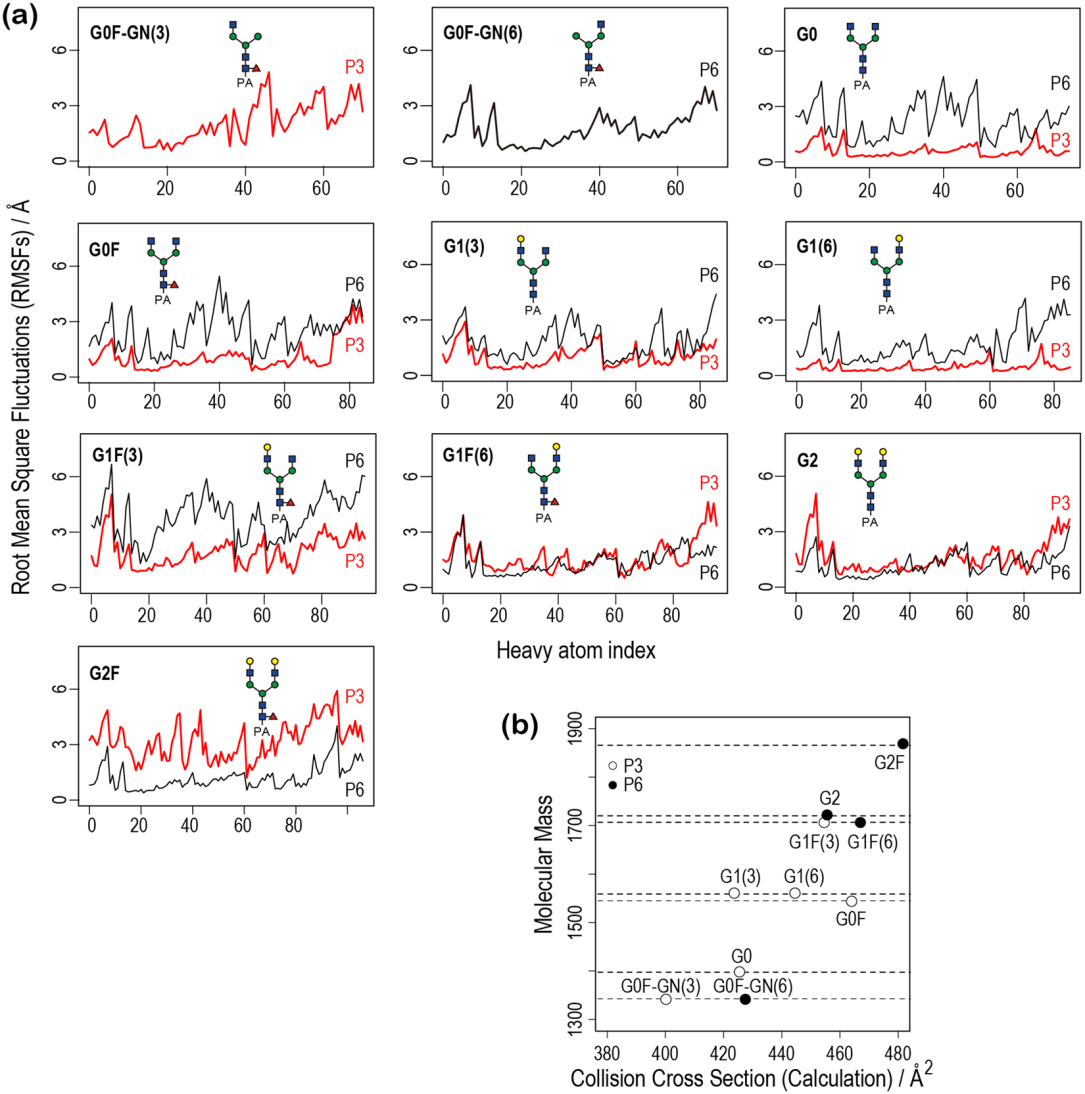
(**a**) RMSFs of heavy atoms calculated for the ten PA-glycans. The protonation states, P3 and P6, are marked using red and black lines, respectively. (**b**) Plot of calculated Collision Cross Sections (CCSs) along molecular mass. P3 and P6 indicate the different protonation states (P3: H^+^ at α1-3 (white), P6: H^+^ at α1-6 branch (black)).

The calculated CCS values for the ten glycans in N_2_ gas as well as in He gas are listed in Table 2. For both P3 and P6 states, they are plotted against experimental values in Fig. 1c. A single protonation state was considered for G0F-GN(3) (core GlcNAc protonated) and for G0F-GN(6) (see Methods for details). Calculated values coincide with experimental ones, with correlation coefficients of 0.90 (P3 state) and 0.89 (P6 state), and average percentage differences of 7.8% (P3 state) and 7.9% (P6 state), respectively (percentage differences were calculated as CCS^calc^−CCS^exp^/CCS^exp^ × 100%). These differences could arise from inaccuracies either in simulations (such as force field parameters) or in experimental calibration. For instance, the use of other calibrants, such as dextran, may improve the accuracy of experimental CCS values. CCS values for the two protonation states of each glycan are different (12 Å^2^ on average), the magnitude depending on type (maximum 22 Å^2^ for G1F(3) and G2, and minimum 5 Å^2^ for G0). The sharp CCS distributions, which were observed experimentally^23^, suggest that dehydration suppresses the dynamics of the sample ion under electrospray ionization conditions. Fig. 2a shows the root mean square fluctuations (RMSFs) of heavy atoms. For most of the glycans, except for G1F(6), G2, and G2F, the P3 state is more constrained than the P6 state. Taking less fluctuating structures, the correlation coefficient between experimental and theoretical values is 0.95. The calculated separations of CCSs for the three isomeric pairs, G0F-GN(3)/G0F-GN(6), G1(3)/G1(6), and G1F(3)/G1F(6), (13 Å^2^–27 Å^2^) well reproduce the experimental data (14 Å^2^–37 Å^2^) (Fig. 2b)^23^. Again consistent with experiment, the glycans with the GlcNAc addition or galactosylation at the α1-6 branch (G0F-GN(6), G1(6), and G1F(6)) have a larger cross section than corresponding isomers (G0F-GN(3), G1(3), and G1F(3)).

**Table 2:** Calculated collision cross sections (in Å^2^) in N_2_ and He drift gas for each of two protonation states, P3 and P6, of ten PA-glycans. The experimental values in the presence of N_2_ drift gas are also listed. The percentage difference between the experimental and calculated CCS in N_2_ drift gas (%diff = CCS^calc^ − CCS^exp^/CCS^exp^ × 100%) are given in parenthesis.

	Experiment	Calculated CCS in N_2_ gas		Calculated CCS in He gas	
		P3	P6	P3	P6
G0F-GN(3)^a^	371	400 (7.8)	—	279	—
G0F-GN(6)	388	—	427 (10.1)	—	296
G0	402	425 (5.7)	420 (4.5)	298	292
G0F	431	464 (7.7)	447 (3.7)	328	315
G1(3)	391	423 (8.2)	436 (11.5)	297	309
G1(6)	428	444 (3.7)	450 (5.1)	313	318
G1F(3)	425	454 (6.8)	476 (12.0)	322	338
G1F(6)	440	475 (8.2)	467 (6.4)	340	336
G2	417	477 (14.4)	455 (9.1)	340	325
G2F	442	475 (7.5)	481 (8.8)	342	350

^a^The core GlcNAc, rather than that of the α1-3 branch, was protonated.

#### CCS distribution and conformational ensemble

**Figure 3:**
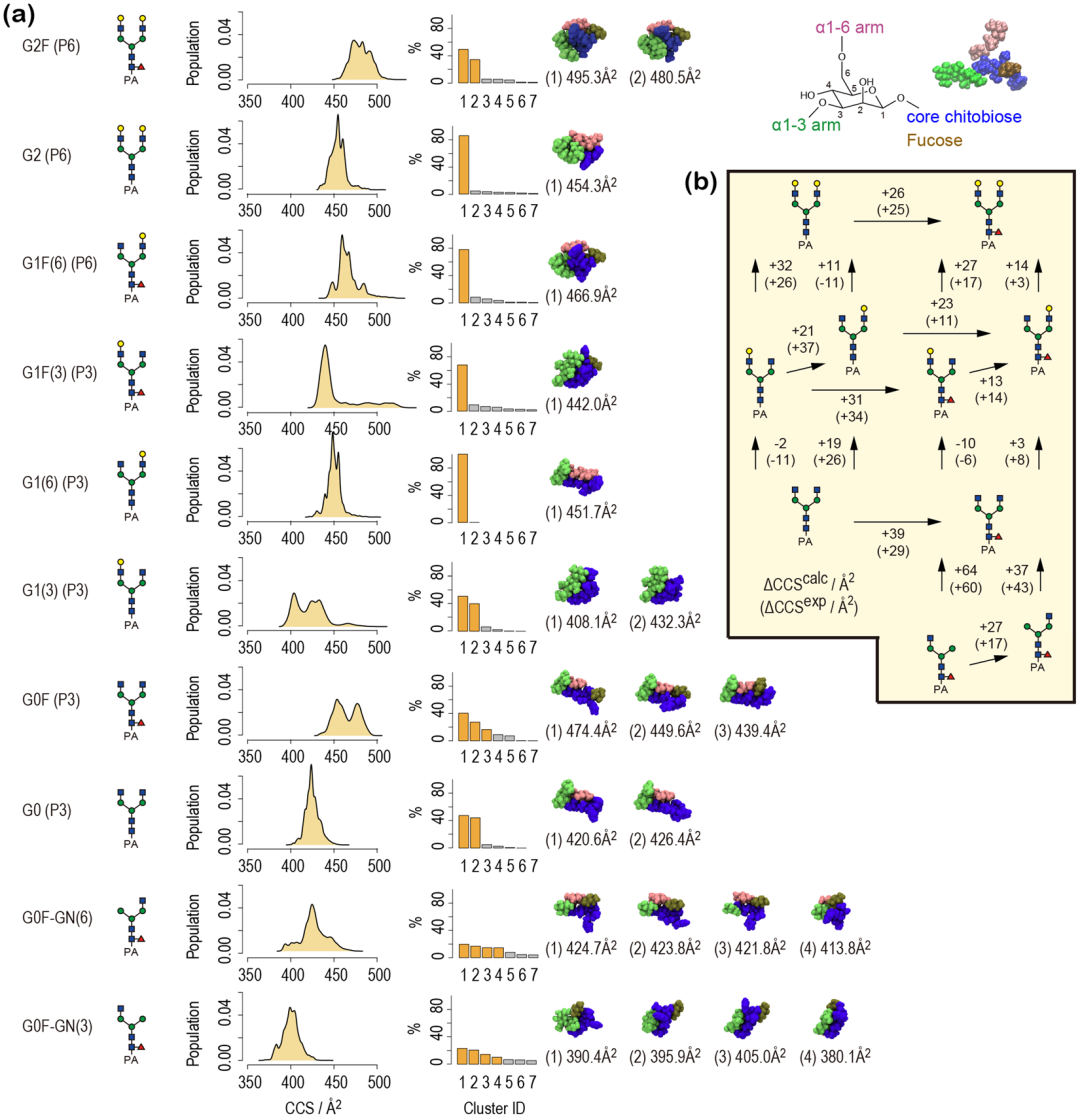
(**a**) CCS distributions, cluster populations that were obtained from k-means clustering using MMTSB toolset (threshold: RMSD = 2.5 Å), and major conformers making up more than 10% of the population (orange bars). The CCS values of major conformers are also given. Blue, green, red, and gold colors for the core chitobiose, α1-3 arm, α1-6 arm, and fucose residue respectively. (**b**) Differences in CCS values (ΔCCS^calc^) are listed with the corresponding values from experiment (ΔCCS^exp^).

**Figure 4:**
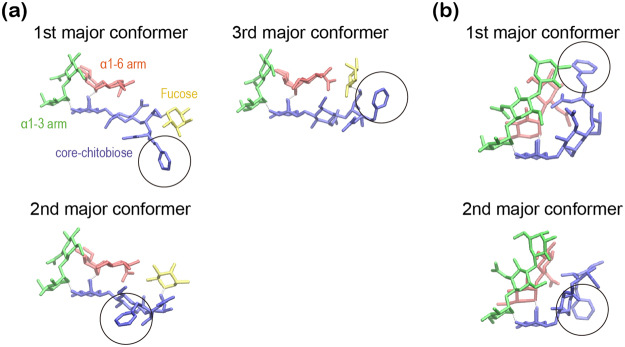
Comparison of major conformers: (**a**) three major conformers in G0F and (**b**) two major conformers in G1(3). Blue, green, red, and yellow colors for the core chitobiose, α1-3 arm, α1-6 arm, and fucose residue, respectively. The pyridylamino (PA) group is marked by a black circle.

**Figure 5:**
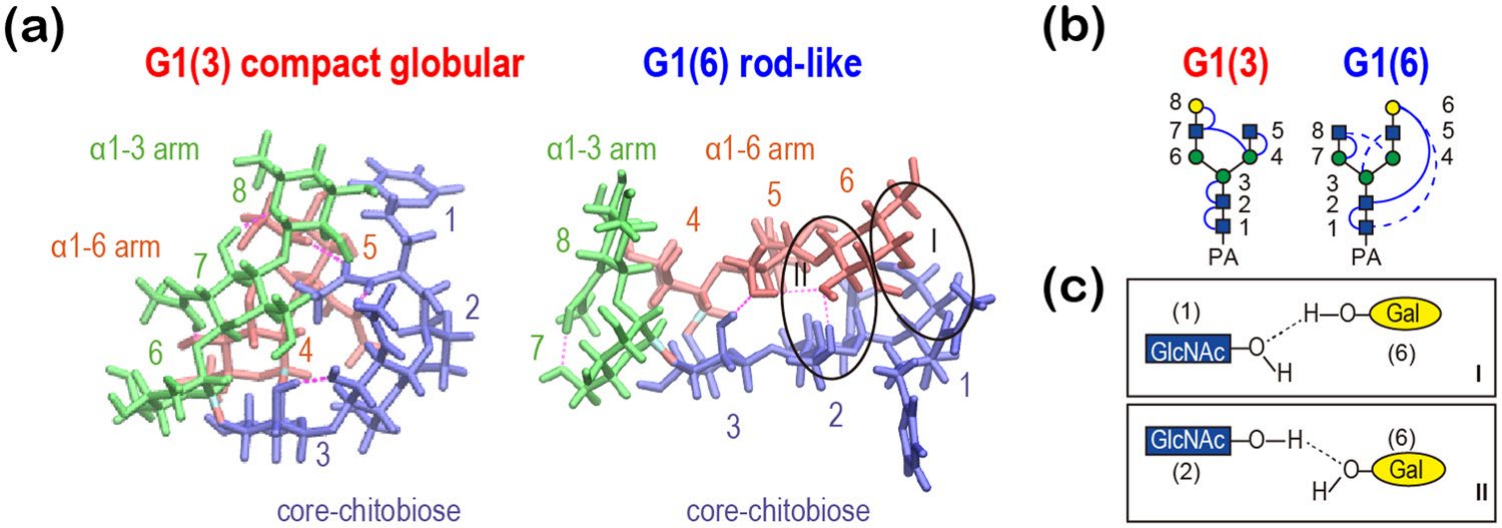
(**a**) Molecular structures of compact globular and rod-like “backfolding” forms. (**b**) Schematic illustration of H-bond networks (solid line for >70% and dashed line for >50% probability). The geometric definition was used to identify H-bonds: R_XY_ < 3.5 Å and θ_HXY_ < 30°, where R_XY_ is the distance between heavy atoms X and Y, and θ_HXY_ is the angle between X–H bond and X–Y vectors. (**c**) Key inter-arm H-bonds in “backfolding” structure (H-bond with the first GlcNAc residue (I) and H-bonds with the second GlcNAc residue (II)).

We examined the calculated CCS distributions of the ten PA-glycans in terms of their conformational ensembles (see Methods for details). We also performed k-means clustering using the MMTSB toolset^37^ (threshold: the root mean square deviation (RMSD) = 2.5 Å) and CCSs were calculated for each cluster. The results are summarized in Fig. 3. We found that the CCS distributions are relatively narrow (within the range of 50 Å^2^) and mostly unimodal. This implies that CCS reflects a few distinct major conformers, and, consistent with this, only one or two clusters have a large population size. Other IM-MS studies on similar N-glycans also reported that a single conformer dominates^29,30^. Different orientation of the core chitobiose, particularly the PA group, gives a bimodal character to the CCS distributions of G1(3) and G0F (Fig. 4). This contrasts with the unimodal character in the experimentally observed arrival times of G1(3) and G0F (Fig. 1b). The discrepancy could arise because either two conformers interconvert in the experimental timescale or there is an inaccuracy of the force field parameters for the PA group. The major conformations of the ten N-glycans are classified into two folded forms described as compact globular and rod-like shapes (Fig. 5). In the compact globular shape, three arms (Manα1-3, Manα1-6, and core chitobiose) symmetrically bend over the central mannose at the branching point. On the other hand, in the rod-like shape, the Manα1-6 arm preferentially undergoes “backfolding” to the core chitobiose. The glycans having the α1-3 arm longer than the α1-6 arm (G0F-GN(3), G1(3), and G1F(3)) have compact globular shapes (small CCSs), while other glycans favor rod-like shapes (large CCSs). An earlier study on doubly sodiated N-glycans showed that the backfolding of the α1-6 arm is commonly observed in the lowest energy conformers and folding of α1-3 arm leads to small CCSs^30^. Note that the CCS values of rod-like conformers change monotonically upon increasing the molecular mass by adding GlcNAc or Gal residues, implying that the folding of the α1-3 arm causes an unexpected change in CCS values. Further elaborated modelling of proton positions may be required to resolve the multiple features of glycans more accurately.

#### Difference in hydrogen bonding pattern

**Figure 6:**
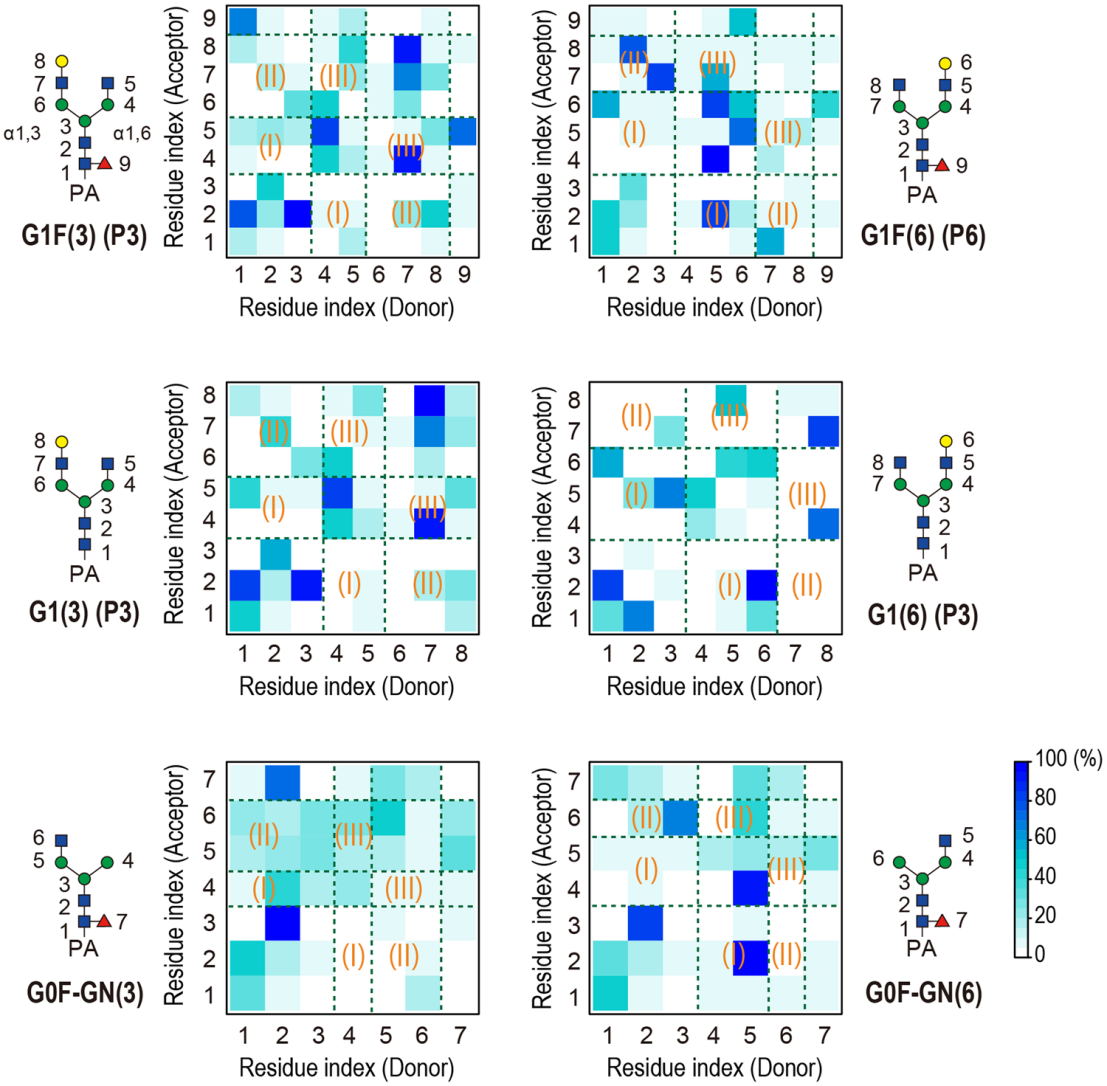
Residue-residue H-bond maps calculated for three pairs of isomeric PA-glycans with selected protonation states. The geometric definition was used to identify H-bonds: R_XY_ < 3.5 Å and θ_HXY_ < 30°, where R_XY_ is the distance between heavy atoms X and Y, and θ_HXY_ is the angle between X–H bond and X–Y vectors. The regions of inter-branch interactions are highlighted with Greek numbers (I: core chitobiose-α1-6 branch, II: core chitobiose-α1-3 branch, III: α1-6 branch-α1-3 branch).

We analyzed H-bond interactions in G1(3) and G1(6), where one galactose is added to G0 (Fig. 5). We used 2,500 snapshots from the trajectory of each system. The following geometric definition was used to define H-bonds: R_XY_ < 3.5 Å and θ_HXY_ < 30°, where R_XY_ is the distance between the heavy atoms X and Y, and θ_HXY_ is the angle between the X–H bond and the X–Y vectors. The residue-residue H-bond maps calculated for all isomeric pairs are shown in Fig. 6. In G1(3), which has the compact globular shape, H-bonds are frequently observed between intra-arm proximal residues. In contrast, we found high probabilities of H-bond interactions between distal residue pairs of the α1-6 arm and the core chitobiose, e.g. 2:GlcNAc—5:Gal (82%) and 1:PA-GlcNAc—6:Gal (52%) in G1(6). In all isomeric glycans, the compact globular shapes have less core-α1-6 H-bonds, while the total number of H-bonds is almost preserved (Table 3). Thus, the separation of CCS values is a result of different H-bonding patterns in the isomers. In the studied N-glycans, the flexible Manα1-6 linkage facilitates backfolding of the α1-6 arm to maximize stabilization via H-bonds. When the α1-3 arm is longer than the α1-6 arm, the rather rigid Manα1-3 linkage also undergoes backfolding because the resultant H-bond stabilization compensates for the distortion penalty. Interestingly, our previous simulation showed that N-glycans in solution favor the backfolding structures^38^.

**Table 3:** Differences in CCS (Å^2^) and H-bonds between rod-like and globular shapes (X_globular_ − X_rod_; X = CCS, Average number of H-bonds, and Percentage of each type of H-bond). The experimental CCS values are given in parentheses.

	G0F-GN	G1	G1F
CCS (exp.)	−27 (−17)	−21 (−37)	−12 (−15)
Av. Num. of H-bonds	2	1	0
% core-α1-6 H-bonds	−18	−28	−14
% core-α1-3 H-bonds	15	9	−11
% α1-3-α1-6 H-bonds	4	5	11
% Intra-arm H-bonds	−1	13	14

## Conclusions

MD simulations have revealed that the protonation state as well as the conformational ensemble are important determinants for CCSs of gas-phase N-glycans. In the studied N-glycans, single conformers of either globular or rod-like shapes dominate and their CCSs are sufficiently different for identification of isomeric N-glycan structures. The results provide a physicochemical basis for IM-MS separation of complex glycan isomers as well as facilitating interpretation of experimental spectra. Furthermore, CCSs of new glycan structures can now be computationally predicted by combining simulations and MOBCAL prediction of the spectra. Recent developments in the field of CCS computations, such as IMoS^39,40^, make accurate CCS calculations for appreciably larger polyatomic ions feasible. Combining CCSs with *ab initio* MD simulation even allows us to accurately model protonation states^41^. The present work lays the foundation for combining IM-MS and simulation to accurately identify complex glycan structures in living organisms.

## Methods

### Model preparation

We modeled 2H^+^ adducts of pyridylamino (PA)-glycans ([M + 2H]^2+^) that were studied in our previous work^23^. The positions of attached protons are not known experimentally. Considering that the amide group is more readily protonated than the hydroxyl group^31^ and that two protons repel each other due to electrostatic repulsion, we built two models, both of which have one H^+^ on PA and a second H^+^ on the distal N-acetylglucosamine (GlcNAc) of either α1-3 (P3) or α1-6 branch (P6). The structures of protonated GlcNAc (PGLN) and pyridylaminated GlcNAc (PA) are shown in Supplementary Figure S1.

In PGLN, both N-H and C=O groups could be protonated. In order to determine the protonation site, proton affinities were estimated for both groups using quantum chemical calculations at the B3LYP/6-31 G(d,p) level. The proton affinity was estimated by subtracting the electronic energy of protonated PGLN from that of neutral PGLN. The geometries of the two forms (protonated and neutral PGLN) were optimized both in gas-phase and in aqueous solution. The effect of solvent was taken into account by using the polarized continuum model^42,43^ (dielectric constant of 78.35). All calculations were performed using the Gaussian09 program package^44^. The results show that the C=O group has a higher proton affinity than the N-H group both in the gas-phase (219.7 kcal/mol for N-H and 229.6 kcal/mol for C=O) and in solution (262.3 kcal/mol for N-H and 271.2 kcal/mol for C=O). Therefore, we decided to protonate the C=O group for PGLN. This proton position is supported by the estimated pKa using the Marvin pKa plugin from ChemAxon (Marvin 16.6.27.0 ChemAxon Ltd. http//www.chemaxon.com) (Supplementary Figure S1). The use of such a pKa program helps to theoretically model proton positions that are not known experimentally, and may be followed by more expensive quantum chemical calculations to elaborate the model.

Note that we considered a single protonation state for G0F-GN(3), in which the core GlcNAc was protonated. Protonation of GlcNAc of the α1-3 branch in G0F-GN(3) gives a CCS value significantly larger than that of G0F-GN(6). This is inconsistent with experimental evidence^23^, which indicates that the CCS of G0F-GN(3) is smaller than that of G0F-GN(6). In G0F-GN(3), the length of the flexible α1-6 branch is exceptionally short compared to the other N-glycans studied, and this could increase surface exposure of the core GlcNAc and the possibility of protonation. Indeed, protonation of the core GlcNAc gives a much better correlation between calculated and experimental CCSs.

### Force field parameterization

The parameters for PGLN and PA were newly developed based on the CHARMM36 carbohydrate^32,45,46^ and CHARMM General Force Field^47,48^. In order to preserve consistency with existing force fields, we assigned available parameters to fragments (GlcNAc for PGLN and D-Threitol, Alanine dipeptide, Morpholine, and 2-acetamide pyridine for PA) and determined the partial atomic charges and dihedral angle parameters only for atoms proximal to the added proton as shown in Supplementary Figure S2.

For the charges of PGLN, the CHARMM36 values for GlcNAc were used for the glucose moiety. The charges of the acetamide moiety were derived from the electrostatic potential fit (ESP) charges obtained from the quantum chemistry calculation of acetamide using the Merz-Singh-Kollman scheme^49,50^ at the MP2/6-31 G(d) level. Similarly, the CHARMM36 charges for D-threitol and the CHARMM General Force Field charges for the alanine dipeptide, morpholine, and 2-acetamide pyridine were assigned to each of the four fragments for PA. Then, we modified the charges around the added proton (NH_2_^+^ group) according to the ESP charges calculated for PA. Note that we manually modified some values in order to preserve a total charge of +1. The final charges for the two residues are given in Supplementary Table S1.

For dihedral angle parameters, we optimized two missing parameters, χ0 in PA and χ3 in PGLN, to reproduce the *ab initio* potential energy surfaces (PESs) of the model compounds shown in Supplementary Figure S3. The *ab initio* PESs were obtained at the MP2/cc-pVTZ//MP2/6-31 G(d) level. The PES was scanned in 30° increments for the corresponding dihedral angles. The parameters were determined by fitting the empirical PES to the entire range of *ab initio* PES using the fit_dihedral.py script^51^, with the optimized parameters listed in Supplementary Table S2. The newly determined parameter sets well reproduce the *ab initio* PESs for the rotations of χ0 and χ3 in the model compounds, as shown in Supplementary Figure S3. Note that the *ab initio* PESs for the rotations of χ1 and χ2 angles of PGLN were reasonably reproduced using the default parameters, although the added proton could affect these angles significantly.

### Molecular dynamics simulations

The replica-exchange molecular dynamics (REMD) simulations^26^ were performed for 18 PA-glycan systems in total: A single protonation state for G0F-GN(3) and G0F-GN(6), and two protonation states for the rest of the eight PA-glycans. The in-house interface program (REIN: Replica-Exchange Interface version 0.1)^35^ was used for REMD simulations. The NAMD program^52^ was used for each MD simulation inside of REIN. The initial configurations were constructed using the GLYCAM web portal (Glycam Biomolecule Builder, http://glycam.org)^53^. All the simulations were carried out in vacuum. The Langevin dynamics method was used to maintain the temperature (damping constant = 5 ps^−1^). Nonbonded interactions were calculated without any truncation, using a large cutoff distance (50 Å). The bonds involving hydrogen atoms were kept rigid using SHAKE. A time step of 2 fs was used in the simulations. Each system was equilibrated using a constant temperature MD simulation at 300 K before the REMD simulation. The final snapshots were used to initiate the subsequent REMD simulations. Sixteen replicas were used to cover the temperatures ranging from 300 K to 1,058 K. To keep the chair conformation of the pyranose rings at high temperatures, six dihedral restraints were applied to the ring. Each replica was simulated for 50 ns with replica exchange trials at every 2 ps. The total simulation time was 0.8 μs (50 ns × 16 = 800 ns) for each system. Only the trajectory at 300 K was used for analyses. The convergence of simulations was assessed by checking the acceptance ratio, replica exchanges, and the relative population sizes of conformers. In Supplementary Figure S4, we show data obtained for G1F(3) as an example. The acceptance ratio is 28% on average. We confirmed random walks both in the replica space and temperature space. We plotted the relative population size of each conformer having different α1-6 linkage orientations. Each population changes drastically in the first 10 ns and converges reasonably within 50 ns. We obtained similar results for the other glycans.

### Calculation of collision cross section (CCS)

CCS values in N_2_ drift gas were calculated using the trajectory method^27,33^. We used the modified version of the MOBCAL program and a newly parametrized algorithm of the N_2_-based trajectory method^34^. The structures from REMD simulations were used for CCS calculations without optimization. In the MOBCAL program, we used the same partial charges as for the REMD simulations that we parametrized to reproduce the results of quantum chemical calculations as described in “Force field parametrization”. Since the trajectory method is computationally very expensive, we used a conformational clustering to reduce the number of CCS calculations. First, we obtained representative conformers by the k-means method (threshold RMSD: 2.5 Å) implemented in the MMTSB Tool Set^37^. We obtained 13 clusters on average for each glycan. Then, the CCS value was calculated for the center structure of each cluster. We also performed clustering analysis with a small threshold (RMSD: 0.5 Å) to obtain a fine distribution of CCSs. This produces a maximum of 2,625 clusters and the CCS value was calculated for the center structure of each cluster. Finally, the CCS value of each center structure was weighted by multiplying the number of structures involved in the corresponding cluster, giving a fine distribution of CCSs as shown in Fig. 3. In the CCS calculation, we set the number of integration points to 25 in the Monte Carlo integration of the impact factor and orientation^54^. The CCS values in He drift gas were also calculated for comparison.

## Electronic supplementary material

Supplementary Information
