# Supplementary material for: Characterization of Conformational Ensembles of Protonated N-glycans in the Gas-Phase
Source: Sci Rep. 2018 Jan 26;8:1644. doi: 10.1038/s41598-018-20012-0 (PMC5786100; doi:10.1038/s41598-018-20012-0)
Supplement: Supplementary file 1 — Supplementary Information [file 41598_2018_20012_MOESM1_ESM.pdf]

Supplementary Information on "Characterization of Conformational Ensembles of Protonated N-glycans in the Gas-Phase"

Suyong Re,[†] Shigehisa Watabe,[†,§] Wataru Nishima,[†,‡] Eiro Muneyuki,[§] Yoshiki Yamaguchi,[¶] Alexander D. MacKerell Jr.[#] and Yuji Sugita[†,‡,*]

[†]RIKEN Theoretical Molecular Science Laboratory, 2-1 Hirosawa, Wako, Saitama 351-0198, Japan. [‡]RIKEN iTHES, 2-1 Hirosawa, Wako, Saitama 351-0198, Japan. [§]Graduate School of Science and Engineering, Chuo University, 1-13-27, Kasuga, Bunkyo-ku, Tokyo 112-8551, Japan. [¶]Structural Glycobiology Team, Systems Glycobiology Research Group, RIKEN Global Research Cluster, 2-1 Hirosawa, Wako, Saitama 351-0198, Japan. [#]Department of Pharmaceutical Sciences, School of Pharmacy, University of Maryland, Baltimore, Maryland 21201, U. S. A.

* Corresponding Author: sugita@riken.jp (Y. S.).

Supplementary Tables

Table S1. Atomic charges for the two residues, PGLN and PA, used in the simulation.

RESI PGLN				RESI PA			
	Name	Type	Charge		Name	Type	Charge
ATOM	H01	HCP1	0.420	ATOM	C01	CG324	-0.044
ATOM	O02	OC311	-0.650	ATOM	H02	HGA2	0.100
ATOM	C03	CC3162	0.340	ATOM	O03	OC311	-0.650
ATOM	H04	HCA1	0.090	ATOM	C04	CC312	0.140
ATOM	O05	OC3C61	-0.400	ATOM	H05	HCA1	0.100
ATOM	C06	CC3163	0.110	ATOM	C06	CC322	0.050
ATOM	H07	HCA1	0.090	ATOM	H07	HCA2	0.100
ATOM	C08	CC321	0.050	ATOM	H08	HCA2	0.100
ATOM	H09	HCA2	0.090	ATOM	O09	OC311	-0.650
ATOM	H10	HCA2	0.090	ATOM	H10	HCP1	0.430
ATOM	O11	OC311	-0.650	ATOM	C11	CC312	0.140
ATOM	H12	HCP1	0.420	ATOM	H12	HCA1	0.100
ATOM	C13	CC3161	0.140	ATOM	C13	CC322	0.050
ATOM	H14	HCA1	0.090	ATOM	H14	HCA2	0.100
ATOM	O15	OC311	-0.650	ATOM	O15	OC311	-0.650
ATOM	H16	HCP1	0.420	ATOM	H16	HCP1	0.430
ATOM	C17	CC3161	0.140	ATOM	C17	CG311	0.070
ATOM	H18	HCA1	0.090	ATOM	H18	HGA1	0.100
ATOM	O19	OC311	-0.650	ATOM	N19	NG2S1	-0.470
ATOM	H20	HCP1	0.420	ATOM	H20	HGP1	0.310
ATOM	C21	C21	0.110	ATOM	C21	CG2O1	0.510
ATOM	H22	HCA1	0.090	ATOM	O22	OG2D1	-0.510
ATOM	N23	N23	-0.210	ATOM	C23	CG331	-0.270
ATOM	H24	H24	0.360	ATOM	H24	HGA3	0.100
ATOM	C25	C25	0.570	ATOM	H25	HGA3	0.100
ATOM	O26	O26	-0.450	ATOM	H26	HGA3	0.100
ATOM	C27	C27	-0.650	ATOM	O27	OC311	-0.650
ATOM	H28	HCA3	0.220	ATOM	H28	HCP1	0.430
ATOM	H29	HCA3	0.240	ATOM	N29	NG3P2	0.033
ATOM	H30	HCA3	0.240	ATOM	H30	HGP2	0.133
ATOM	H31	HCP1	0.480	ATOM	H31	HGA2	0.100
				ATOM	C32	CG2R61	0.805
				ATOM	C33	CG2R61	-0.115
				ATOM	N34	NG2R60	-0.600
				ATOM	C35	CG2R61	-0.115
				ATOM	H36	HGR61	0.115
				ATOM	C37	CG2R61	0.180
				ATOM	C38	CG2R61	-0.115
				ATOM	H39	HGR61	0.115
				ATOM	H40	HGR62	0.120
				ATOM	H41	HGR61	0.115
				ATOM	H42	HGP2	0.133
				ATOM	H43	HCP1	0.430

Table S2. The optimized two missing dihedral angle parameters, $\chi 0$ in PA and $\chi 3$ in PGLN.

```
!V(dihedral) = Kchi(1 + cos(n(chi) - delta))
       !Kchi: kcal/mole
       !n: multiplicity
       !delta: degrees
```

atom types				Kchi	n	delta
(x0)						
CG2R61	CG2R61	NG3P2	CG324	0.5900	1	180.00
CG2R61	CG2R61	NG3P2	CG324	2.2100	2	180.00
CG2R61	CG2R61	NG3P2	CG324	0.0800	3	0.00
CG324	NG3P2	CG2R61	NG2R60	0.7000	1	180.00
CG324	NG3P2	CG2R61	NG2R60	1.9000	2	0.00
CG324	NG3P2	CG2R61	NG2R60	0.4200	3	0.00
HGP2	NG3P2	CG2R61	NG2R60	1.3700	1	180.00
HGP2	NG3P2	CG2R61	NG2R60	0.9400	2	0.00
HGP2	NG3P2	CG2R61	NG2R60	0.0800	3	0.00
CG2R61	CG2R61	NG3P2	HGP2	1.1700	1	180.00
CG2R61	CG2R61	NG3P2	HGP2	1.1500	2	180.00
CG2R61	CG2R61	NG3P2	HGP2	0.1800	3	0.00
(x3)						
HCP1	O26	C25	N23	2.0100	1	180.00
HCP1	O26	C25	N23	2.9300	2	180.00
HCP1	O26	C25	N23	0.0200	3	180.00
C27	C25	O26	HCP1	1.7300	1	180.00
C27	C25	O26	HCP1	2.2000	2	180.00
C27	C25	O26	HCP1	0.2200	3	180.00

Supplementary Figures

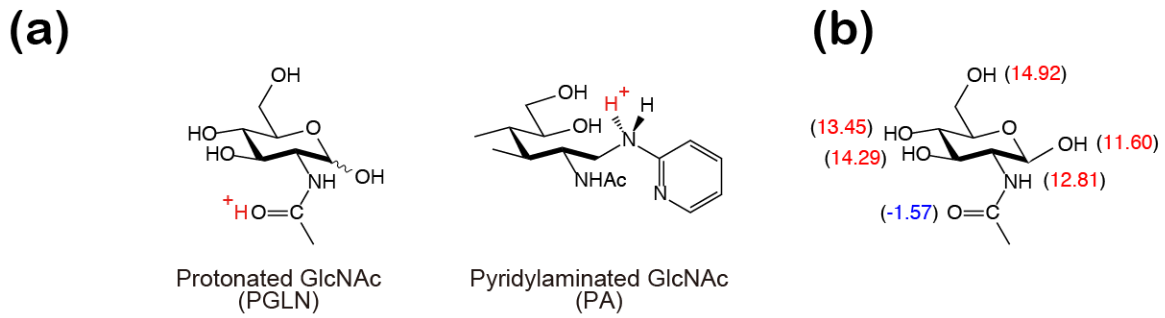

Figure S1. (a) Structures of protonated GlcNAc (PGLN) and pyridylaminated GlcNAc (PA). (b) The pKa values of GlcNAc residue estimated by using the Marvin pKa plugin from ChemAxon. Blue: the acid dissociation constant between the neutral base and its conjugate acid, Red: that between the neutral acid and its conjugate base.

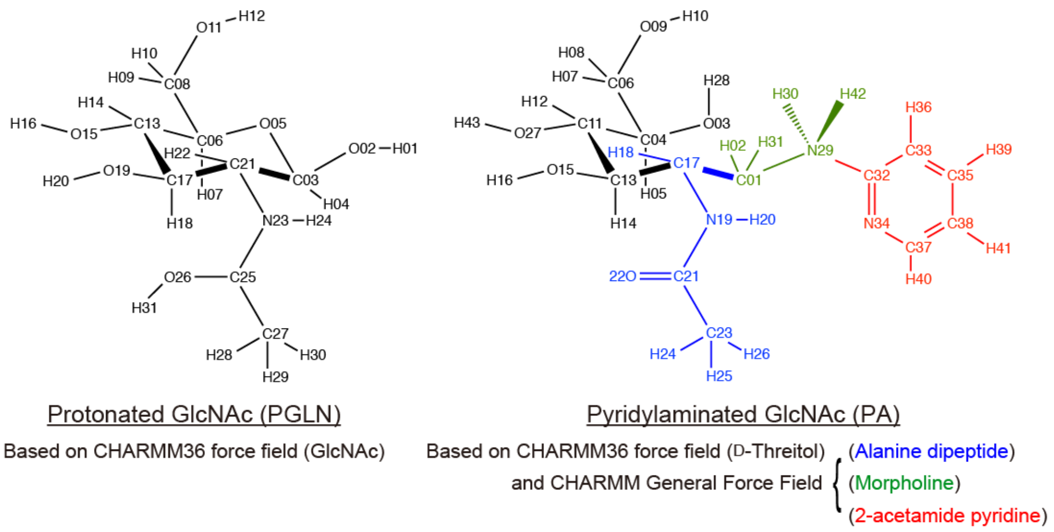

Figure S2. Assignment of available parameters to fragments of protonated GlcNAc (PGLN) and pyridylaminated GlcNAc (PA).

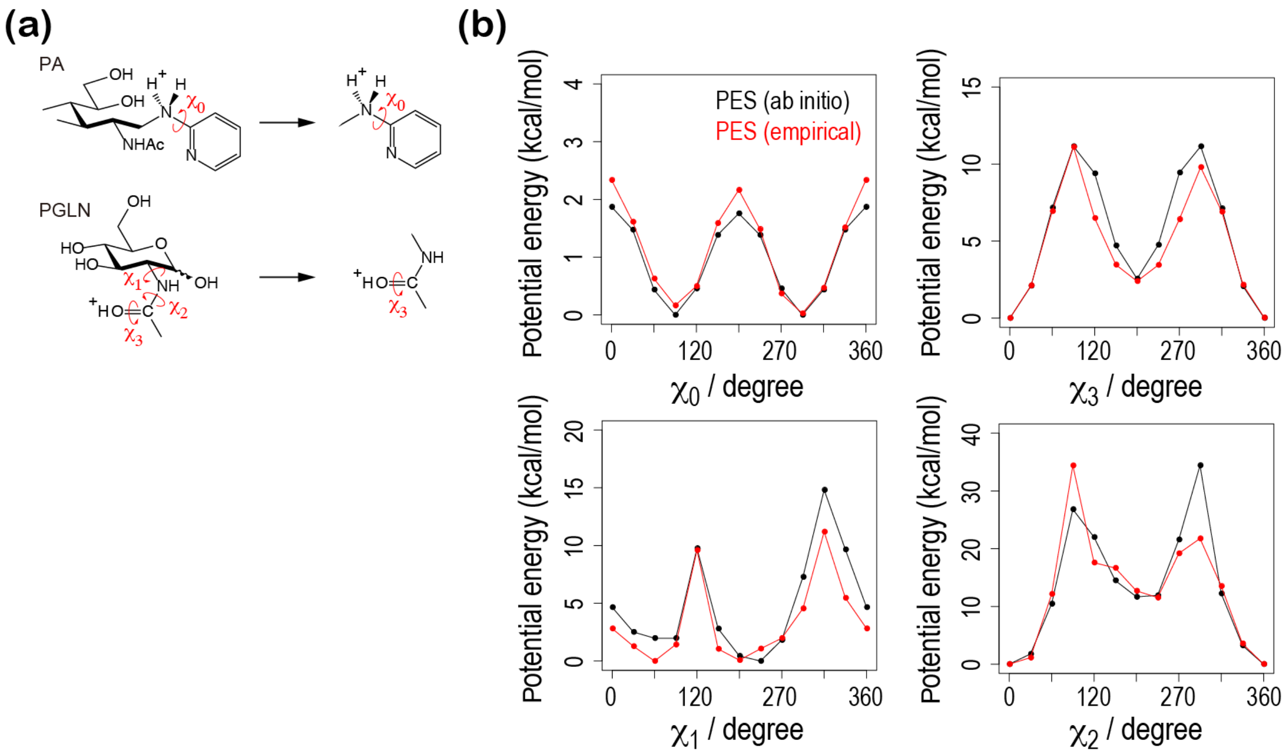

Figure S3. (a) Model compounds used for the parameter fit of protonated GlcNAc (PGLN) and pyridylaminated GlcNAc (PA). (b) *ab initio* and empirical potential energy surfaces (PESs) for the rotations of $\chi0$ in PA as well as $\chi1$, $\chi2$, and $\chi3$ angles of PGLN.

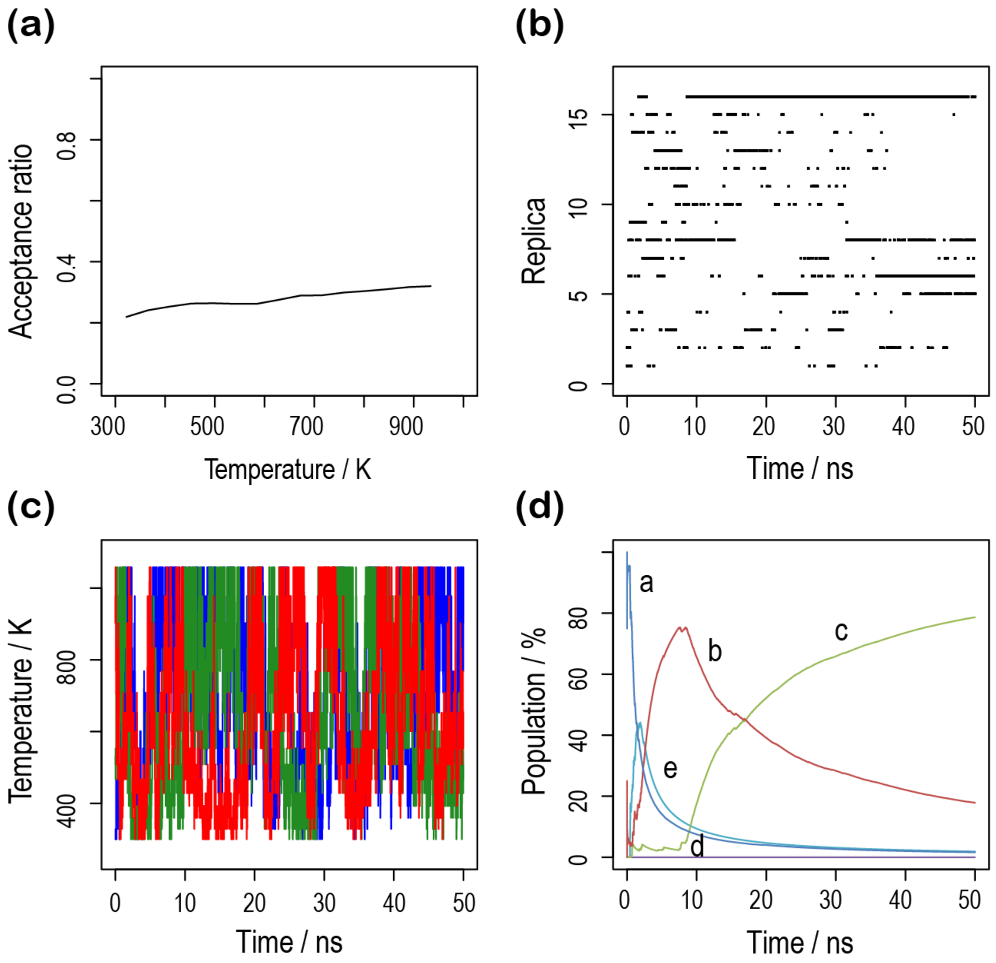

Figure S4. (a) The acceptance ratios of replica exchanges between adjacent pairs at each temperature in the simulation of G1F(3) (P3). (b) Replica exchange at temperature of 300 K and (c) temperature exchanges of three arbitrary chosen replicas (replica 1 (blue), 15 (green), and 17 (red)). (d) Convergence of the population of each conformer (a: $-60° \leq \psi < 75°$ and $-60° \leq \omega < 120°$, b: $-75° \leq \psi < 135°$ and $-60° \leq \omega < 120°$, c: ($-60 > \psi$ or $135° \leq \psi$) and $-60° \leq \omega < 120°$, d: $-60° \leq \psi < 135°$ and ($\omega < -60°$ or $120° \leq \omega$), e: ($-60° > \psi$ or $135° \leq \psi$) and ($\omega < -60°$ or $120° \leq \omega$).
